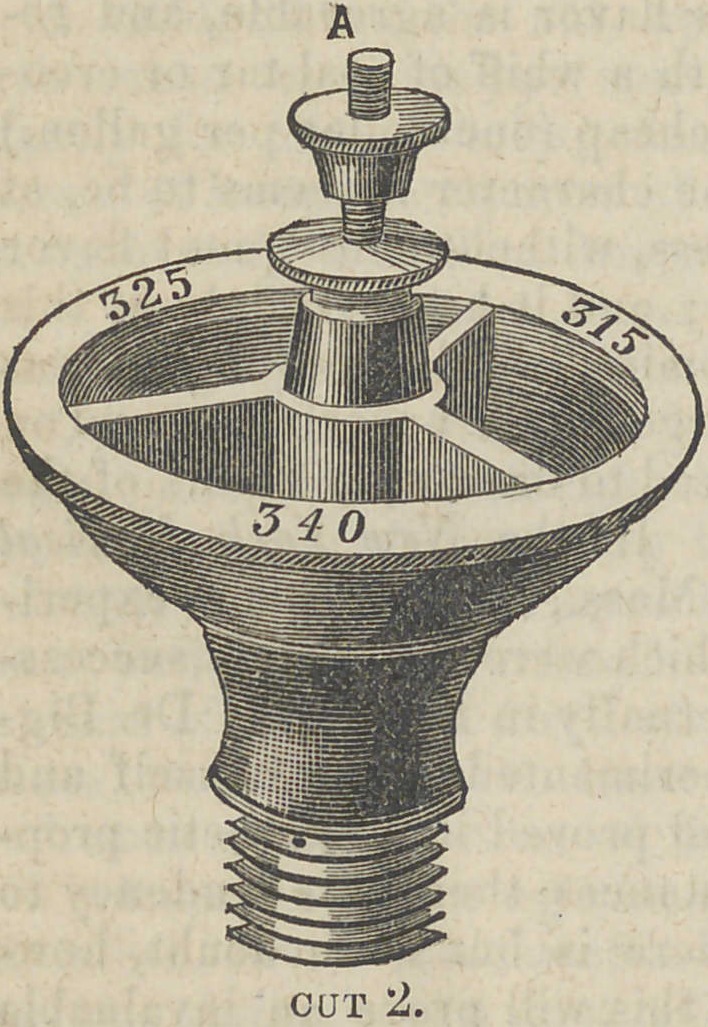# Fusible Gauge

**Published:** 1861-11

**Authors:** 


THE annexed cuts represent "Franklin's Fusible Gauge," to be used on vulcanizers; instead of the ordinary thermometer. The apparatus is very simple, and easily managed by any one who is familiar with vulcanizing machines. It consists of a brass cup, about two inches and a half in diameter, divided into three compartments ; into each of which is placed a fusible alloy, that in each one differing in point of fusibility from the others, one melting at 315°, the next at 325°, the next at 340°.

Either up through the center of the cup, or through the shaft below it, is fixed a safety valve ; the arrangement of this is the same as that used with the common vulcanizers.

We will give the directions for its use in Dr. Franklin's language.

"When the alloy in the compartments marked 315 has become *granular*, the temperature in the vulcanizer is 295 degrees ; as it changes from the *granular* to a *mushy* condition, the heat has increased to 310 degrees. This is the true vulcanizing heat for two and a half hours, and when it has become *fluid*, the heat has increased to 320 degrees, which is fully demonstrated by a comparison with the best Standard Thermometers and Steam Gauges.

"The alloy in the compartment marked 325 begins to be *granular* at 320 degrees, and in that condition a pointed instrument can be forced into the center of the alloy, while the outer portion remains firm. This is the true vulcanizing heat for one and a half hours. This alloy loses its granular condition and becomes *mushy* at 330 degrees, and when *fluid*, the temperature of the vulcanizer is 340 degrees.

"The alloy in the compartment marked 340 is of little practical value except to indicate an extreme degree of heat. It is slightly *granular* at 340, and fluid at 360 degrees."

We have used this guage for a short time, and think it equally as good as the thermometer in all respects, and much better in some, the chief of which is, that it can not get out of repair. All who have used the ordinary thermometer are aware of the liability to breakage, and the risk incurred thereby; with this there is no breakage. The only change that one can at all imagine, would be the change in the composition of the metals occasioned by the frequent melting ; this will, however, be very slight. Dr. Franklin remarks that he has used it daily for eight months, and finds no change in the metals by oxydation ; that being the case, there can be no objection on account of its change, and even if it did change once every six months, so as to destroy its efficiency, one or two cents would supply a new piece of metal. Only one of these is fused at a vulcanizing heat.

It is possible that a current of cold air, or a jet of steam, from the safety valve, might interrupt slightly the perfect action of the heat upon the metals ; that, however, can be guarded so as to occasion no annoyance. Perhaps on account of the escape of steam, it will be better to have the safety valve below the cup, as in cut 1—in that the steam is not so likely to come in contact with the fusible metals. A very little practice with this gauge will enable the workmen to attain the same accuracy as with the most perfect thermometer.

We vulcanize our work in one hour after the alloy marked 315° is perfectly fused ; we run the heat up, however, till the metal in 325° is in a very soft, mushy condition, almost in a fluid state. A little experience will enable any one to use it with perfect accuracy.

The instrument will probably be for sale at all the Dental Depots, but for the present it is furnished by B. W. Franklin, No. 73 Bleeker st., New York. Price $3.00.

In ordering these gauges, it is well to specify the kind of heater upon which they are to be used.

Duplicate pieces of metal are always sent with the gauges.

## Figures and Tables

**Cut 1. f1:**
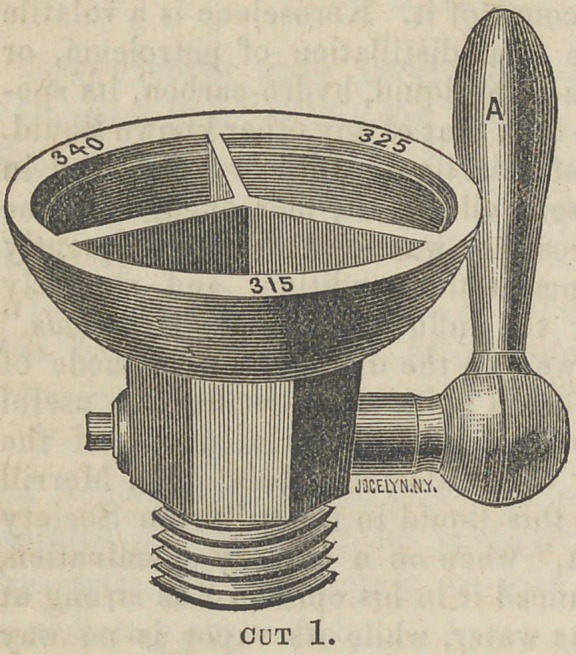


**Cut 2. f2:**